# Effect of different doses of intravenous oxycodone and fentanyl on intubation-related hemodynamic responses: A prospective double-blind randomized controlled trial (CONSORT)

**DOI:** 10.1097/MD.0000000000015509

**Published:** 2019-05-03

**Authors:** Gi-Ho Koh, Ki Tae Jung, Keum Young So, Jong Sik Seo, Sang Hun Kim

**Affiliations:** a Department of Anesthesiology and Pain Medicine, Asan Medical Center, University of Ulsan College of Medicine, Seoul; b Department of Anesthesiology and Pain Medicine, Chosun University Hospital; c Department of Anesthesiology and Pain Medicine, Chosun University School of Medicine; d Department of Medicine, Graduate School of Chosun University, Gwangju, Republic of Korea.

**Keywords:** fentanyl, hemodynamic responses, intubation, oxycodone

## Abstract

**Background:**

Intubation using direct laryngoscopy is a risky and painful procedure that is associated with undesirable hemodynamic changes such as tachycardia, hypertension, and arrhythmia. Recently, intravenous oxycodone was introduced and used for the control of acute postoperative pain and to attenuate intubation-related hemodynamic responses (IRHRs), but there is insufficient information regarding its proper dosage. We investigated the attenuating effects of different doses of oxycodone and fentanyl on IRHRs.

**Methods:**

For calculating oxycodone effective dose (ED_95_), which attenuated all IRHR changes to less than 20% over baseline values in 95% of male patients at 1 minute after intubation, oxycodone 0.1 mg/kg was injected for the first patient 1 hour before intubation, and the next dose for each subsequent patient was determined by the response of the previous patient using Dixon up-and-down method with an interval of 0.01 mg/kg. After obtaining the predictive oxycodone ED_95_, 148 patients were randomly allocated to groups receiving normal saline (group C), oxycodone ED_95_ (group O1), oxycodone 2 × ED_95_ (group O2), or fentanyl 2 μg/kg (group F). We recorded the incidence of “success” as a less than 20% change from baseline values in all IRHRs 1 minute after intubation.

**Results:**

The predictive oxycodone ED_95_ was 0.091 (0.081–0.149) mg/kg. The incidence of “success” was highest in group O2 (75.7%), followed by group O1 (62.2%) and group F (45.9%) with significant differences between the groups (*P* < .001). The systolic, diastolic, mean arterial pressure, and heart rate were not significantly different among groups after administration of either oxycodone or fentanyl. The percentage hemodynamic changes of the group O2 were significantly lower than those of groups F and O1, but the absolute percentage hemodynamic changes were not significantly different among groups F, O1, and O2. The recalculated oxycodone ED_95_ with probit analysis (0.269 mg/kg) was needed to prevent any arterial pressure and heart rate changes.

**Conclusions:**

Oxycodone 0.182 mg/kg is more effective in attenuating all IRHRs than fentanyl 2 μg/kg with safe hemodynamic changes. Further research is required to determine if the recalculated oxycodone ED_95_ (0.269 mg/kg) is also effective and hemodynamically safe for preventing all IRHRs.

## 1 Introduction

Endotracheal intubation using direct laryngoscopy is a risky and painful procedure that causes adverse stimulation and is associated with undesirable hemodynamic changes such as tachycardia, hypertension, and arrhythmia.^[1–3]^ These responses can lead to perioperative complications such as potentially fatal events like cerebral hemorrhage, cardiac arrhythmia, or cardiac failure in patients who have cardiovascular or cerebral disease.^[1]^ Therefore, various pharmaceutical interventions such as opioids, beta-adrenergic blockers, and antihypertensive drugs have been used and studied to attenuate intubation-related hemodynamic responses (IRHRs).^[4]^

Oxycodone (14-hydroxy-7, 8-dihydrocodeinone) is a semisynthetic opioid with an agonistic activity on the mu, kappa, and delta receptors.^[5]^ Oral oxycodone has been used in patients with cancer pain and chronic pain. Recently, intravenous oxycodone was introduced and used for the control of acute postoperative pain,^[6]^ and because intravenous oxycodone provides better postoperative analgesic effect than fentanyl, it can help prevent or attenuate IRHRs.

However, there is insufficient information about the appropriate dosage of oxycodone for this purpose,^[7–9]^ and the pharmacokinetics of oxycodone have a somewhat different effect depending on age and sex.^[10,11]^

Therefore, in this study, we calculated the effective doses (EDs) of intravenous oxycodone that could attenuate all IRHR changes to less than 20% over baseline values in 50% and 95% of male adult patients (ED_50_ and ED_95_, respectively). We then investigated the actual attenuating effects of intravenous oxycodone ED_95_, 2 **×** ED_95_, and fentanyl on these IRHRs.

## 2 Materials and methods

This prospective, randomized, controlled and double-blinded study was approved by the Institutional Review Board of Chosun University Hospital, and registered with the Clinical Research Information Service (CRIS: https://cris.nih.go.kr/, ref: KCT0001751) on January 4, 2016. Written informed consent was obtained from all participants, a legal surrogate, or the parents or legal guardians of participants who were minors. This study was conducted in accordance with the tenets of the Declaration of Helsinki.

We enrolled male patients who were aged 20 to 65 years with an American Society of Anesthesiologists (ASA) physical status I or II, and who were scheduled to undergo elective surgery under general anesthesia. We excluded patients with cardiopulmonary disease, neurovascular disease, brain disease, mental disorders, renal or hepatic function abnormalities, recent medication of monoamine oxidase inhibitor, or a body mass index (BMI) above 25%.

After premedication with intramuscular midazolam (0.05 mg/kg), patients were transported to the operating room. Before the induction of anesthesia, standard patient monitoring devices to obtain electrocardiograms, non-invasive blood pressure, end-tidal partial pressure of carbon dioxide, and peripheral pulse oximetry were applied. A catheter was then installed in the radial artery to continuously monitor blood pressure. Patients were premedicated with ramosetron 0.3 mg for prevention of postoperative nausea and vomiting under preoxygenation with O_2_ 6 L/min via a facial mask.

### 2.1 Part 1

Intravenous oxycodone 0.1 mg/kg was administered slowly over 2 minutes for the first patient 20 minutes before intubation, and anesthesia was induced with propofol 2 mg/kg and rocuronium 0.9 mg/kg 2 minutes before intubation. One minute after intubation, “success” or “failure” was determined based on whether the IRHRs were significantly different from their baseline values. We defined “success” as all changes of systolic, diastolic, or mean arterial pressures (SAP, DAP, and MAP) and heart rate (HR) being less than 20%, and “failure” as any change in these parameters being above 20%. The subsequent patient received the next oxycodone dose, which was decreased or increased with an interval of 0.01 mg/kg by the Dixon up-and-down method, depending on the “success” or “failure” of the previous patient, respectively.^[12,13]^ We performed the Dixon up-and-down method until we obtained 8 crossover points, which were manifested as crossover from “failure” to “success.”^[13]^

### 2.2 Part 2

**Figure 1:**
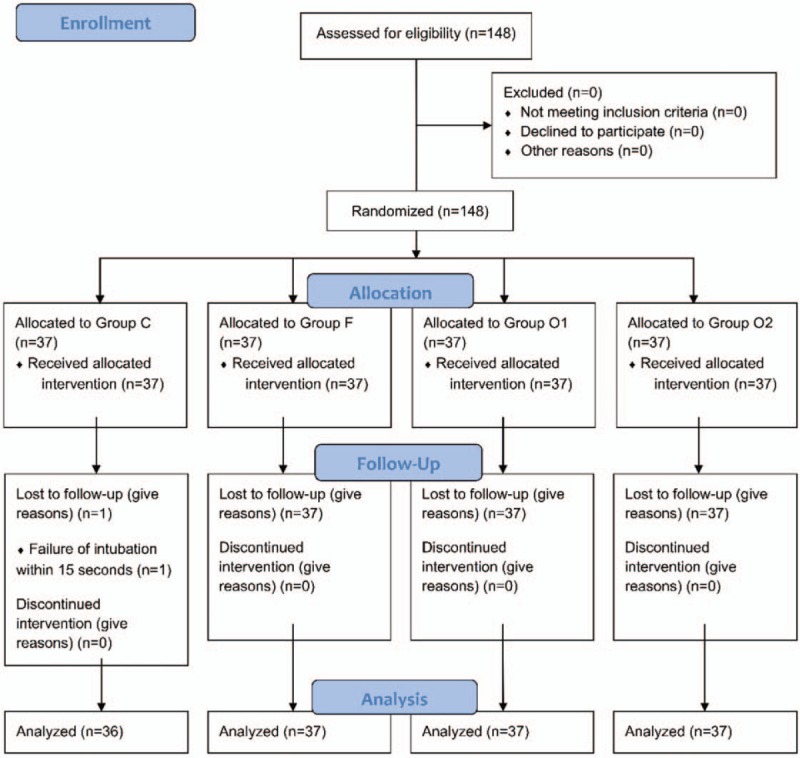
CONSORT diagram in “Part 2.” CONSORT = consolidated standards of reporting trials.

**Figure 2:**
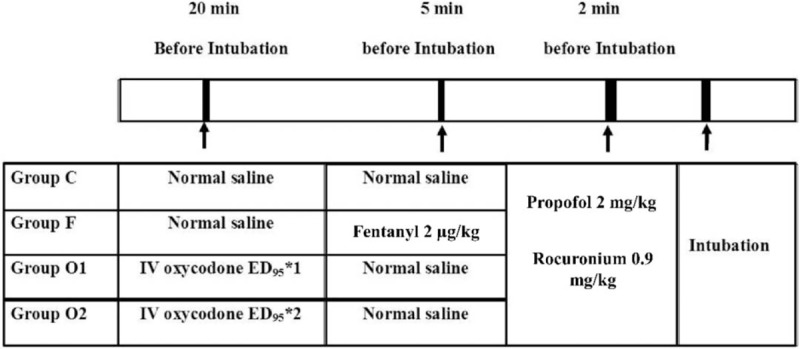
Schematic protocol in “Part 2.” ED_95_: Effective dose of intravenous oxycodone that could attenuate all intubation-related hemodynamic response changes to less than 20% over baseline values in 95% of male adult patients. ED = effective dose.

After obtaining the predictive oxycodone ED_50_ and ED_95_ using a probit regression model in “Part 1,” 148 patients were randomly allocated to one of 4 groups receiving normal saline (group C), oxycodone ED_95_ (group O1), oxycodone 2 **×** ED_95_ (group O2), or fentanyl 2 μg/kg (group F), by using a random numbers table obtained via a computer program (Figs. 1 and 2). Twenty minutes before anesthesia induction, the predictive oxycodone ED_95_ or 2 **×** ED_95_ was administered slowly over 2 minutes, and the 0.9% normal saline was administered in groups C and F. Five minutes before anesthesia induction, fentanyl 2 μg/kg was administered in group F, and 0.9% normal saline was administered in the other groups. Intubation was performed 2 minutes after anesthesia induction with propofol 2 mg/kg and rocuronium 0.9 mg/kg.

We recorded the incidence of “success” or “failure” with the same method as in “Part 1.” We recorded the SAP, DAP, MAP, and HR 20 (baseline values), 5, and 2 minutes before intubation, just before intubation, and 1, 3, 5 minutes after intubation. We also recorded the percentage changes and absolute percentage changes of SAP, DAP, MAP, and HR between the values at baseline and those at 1 minute after intubation. The age, ASA physical status, height, weight, and BMI of each patient were noted.

Patients and investigators were blinded to the study medications. A noninvestigating nurse loaded them into indistinguishable numbered syringes with the same volume (5 mL) and randomized the medications using a random number table.

The primary endpoint was the incidence of “success,” 1 minute after intubation. The secondary endpoints were the changes in SAP, DAP, MAP, and HR values, 1 minute after intubation.

### 2.3 Statistical analysis

For the analysis of Part 2, the necessary sample size for chi-square tests using G∗Power software (ver. 3.1.9.1, Heinrich-Heine-Universität Düsseldorf, Germany) was calculated by taking the level of statistical significance as *α* = 0.05 and *β* = 0.2 and using an expected effect size of 0.28. The effect size was calculated with the expected proportion of “success” for groups C (0.15), F (0.25), O1 (0.25), and O2 (0.35), which was based on a previous pilot study, owing to lack of other evidence for calculating the effect size. The study needed a total of 140 patients, so we enrolled 148 patients, allowing for an assumed 5% dropout rate.

SPSS (Windows ver. 21.0, IBM Corp, Armonk, NY) was used for statistical analysis. All measured values are presented as mean ± standard deviation, mean (95% confidential intervals [CI]) or number (percentage) of patients [n (%)].

In Part 1, ED_50_ of oxycodone was calculated with the mean values of the midpoint dose of all independent pairs of patients who manifested as crossover from “failure” to “success” after 8 crossover points.^[13]^ A probit regression model was used to calculate the predictive oxycodone ED_50_ and ED_95_, and the 95% CIs. In Part 2, the chi-square test was used for analysis of the incidence of “success” and the ASA physical status classification. A 1-way ANOVA followed by Scheffe post-hoc test was used for analysis of hemodynamic responses, percentage changes and absolute percentage changes, ages, height, weight, and BMI. A probit regression model was used to recalculate the predictive oxycodone ED_95_ with data from groups C, O1, and O2. *P* values < .05 were considered statistically significant.

## 3 Results

### 3.1 Part 1

**Figure 3:**
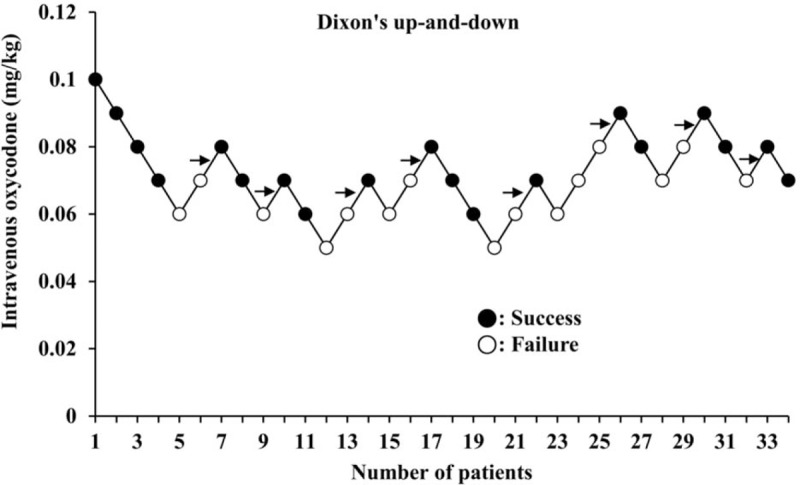
Consecutive dose of intravenous oxycodone for determining the ED_50_ in “Part 1.” The black arrow represents the mean oxycodone doses when crossing from “failure” (white circles) to “success” (black circles). The average of mean oxycodone doses is the ED_50_, which is 0.074 ± 0.008 mg/kg. ED_50_: Effective dose of intravenous oxycodone that could attenuate all intubation-related hemodynamic responses changes to less than 20% over baseline values in 50% of male adult patients. ED = effective dose.

**Figure 4:**
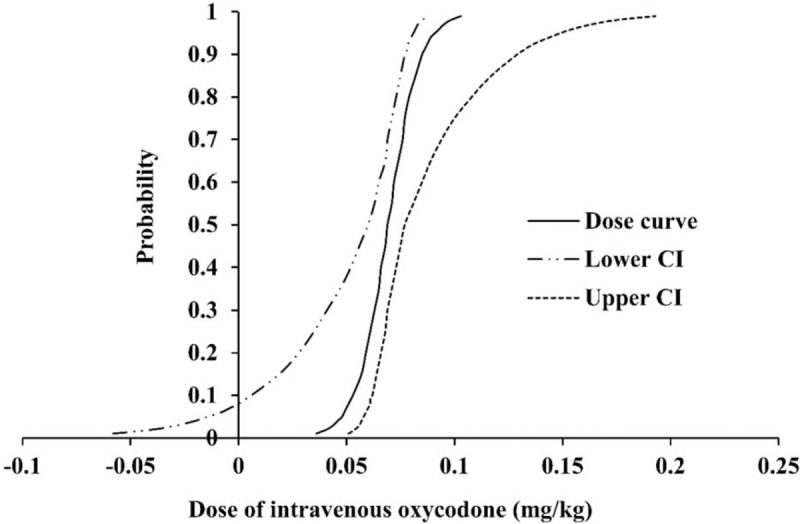
Dose-response curve plotted from probit regression analysis in “Part 1.” The predictive oxycodone ED_50_ and ED_95_ were 0.069 (95% CI, 0.06–0.077) and 0.091 (95% CI, 0.081–0.149) mg/kg. ED_50_, ED_95_: Effective dose of intravenous oxycodone that could attenuate all intubation-related hemodynamic responses changes to less than 20% over baseline values in 50% and 95% of male adult patients, respectively. CI = confidence interval, ED = effective dose.

Thirty-three patients were finally enrolled for the analysis. Oxycodone ED_50_, which was calculated using the Dixon up-and-down method, was 0.074 ± 0.008 mg/kg in male patients (Fig. 3). From the probit regression model, predictive oxycodone ED_50_ and ED_95_ were estimated as 0.069 (95% CI, 0.06–0.077) mg/kg and 0.091 (95% CI, 0.081–0.149) mg/kg in male patients, respectively (Fig. 4).

### 3.2 Part 2

One hundred forty-seven patients were finally enrolled because 1 patient in group C was excluded due to failure of intubation within 15 seconds (Fig. 1). There were no significant differences in age, height, weight, BMI, and ASA physical status among the groups (Table 1).

**Table 1:**
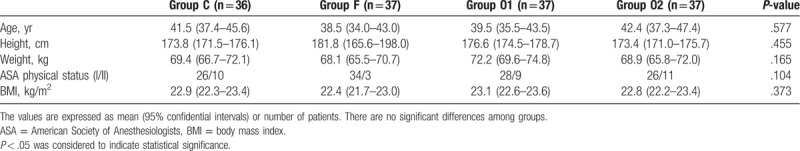
Patient characteristics in “Part 2.”.

The incidence of “success” was significantly different among the groups (Table 2, *P* < .001). It was highest in group O2 (75.7%), followed by group O1 (62.2%) and group F (45.9%), while it was lowest in group C, at 16.7%.

**Table 2:**
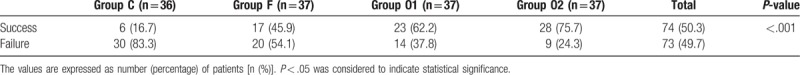
Hemodynamic responses associated with endotracheal intubation in “Part 2.”.

**Figure 5:**
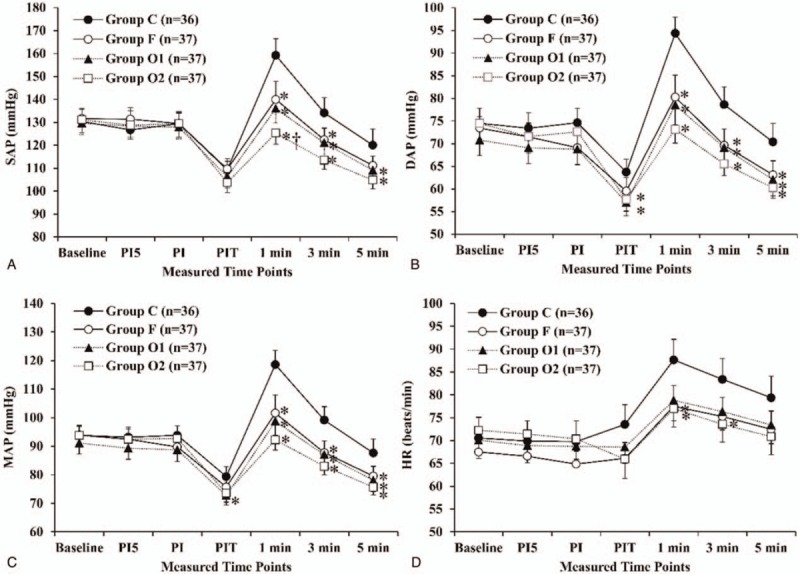
Arterial pressures (systolic [A], diastolic [B], and mean [C]) and heart rate (D) in “Part 2.” One minute after intubation, the SAP, DAP, and MAP were significantly higher in group C compared with the same parameters in groups F, O1, and O2. The SAP of group O2 was significantly lower than that of group F, 1 min after intubation. The HR was significantly lower in groups O2 and F than in group C at 1 min after intubation. The measured time points were 20 (baseline), 5 (PI5), and 2 min (PI) before intubation, just before intubation (PIT), and 1 (1 min), 3 (3 min), 5 min (5 min) after intubation. DAP = diastolic arterial pressure, HR = heart rate, MAP = mean arterial pressure, SAP = systolic arterial pressure.

SAP, DAP, and MAP did not decrease after administration of either oxycodone or fentanyl, but they decreased after propofol injection in all groups (Fig. 5A, B, and C). Groups O1 and O2 showed significantly lower DAP values than that in group C (*P* < .05) after propofol injection, while the SAP and MAP were not significantly different.

SAP, DAP, and MAP increased immediately after intubation, and subsequently declined progressively (Fig. 5A, B, and C). One minute after intubation, SAP, DAP, and MAP were significantly higher in group C than in groups F, O1, and O2 (*P* < .001), and these hemodynamic trends continued until 5 minutes after intubation. They were lowest in group O2, followed by groups O1 and F, but there were no significant differences among these groups, except for SAP. SAP of group O2 was significantly lower than that of group F at 1 minute after intubation (Fig. 5A, *P* = .014).

HRs of all groups did not decrease after administration of oxycodone, fentanyl, or propofol, but they increased immediately after intubation and subsequently declined progressively (Fig. 5D). One minute after intubation, the mean HRs of groups O2 and F were significantly lower than that of group C (*P* < .05), while those of groups O1, O2, and F were not significantly different. Three minutes after intubation, the mean HR of group O2 was significantly lower than that of group C (*P* = .03). HR was not significantly different among the groups at the other time points.

**Figure 6:**
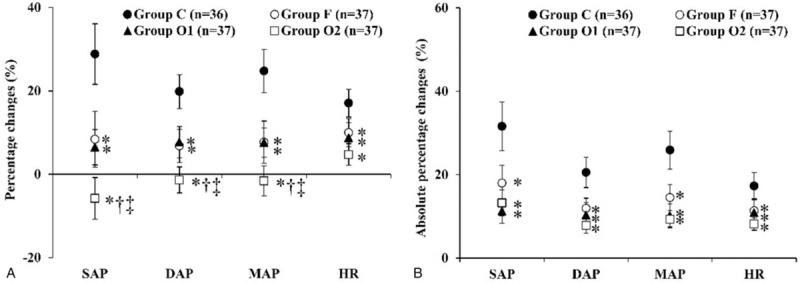
The percentage changes (A) and absolute percentage changes (B) of SAP, DAP, MAP, and HR at 1 min after intubation. The percentage changes and absolute percentage changes of SAP, DAP, MAP, and HR were significantly lower in groups F, O1, and O2, compared with those in group C. The percentage changes of groups O2 was significantly lower than those of groups F and O1, but the absolute percentage changes were not significantly different among groups F, O1, and O2. The absolute percentage changes are the absolute value of each changing percentage (| percentage changes of SAP, DAP, MAP, and HR |). DAP = diastolic arterial pressure, HR = heart rate, IRHR = intubation-related hemodynamic response, MAP = mean arterial pressure, SAP = systolic arterial pressure.

Percentage changes and absolute percentage changes of SAP, DAP, MAP, and HR at 1 minute after intubation were significantly lower in groups F, O1, and O2, compared with those of group C (Fig. 6A and B). Percentage changes of group O2 were significantly lower than those of groups F and O1, but the absolute percentage changes were not significantly different among groups F, O1, and O2.

**Figure 7:**
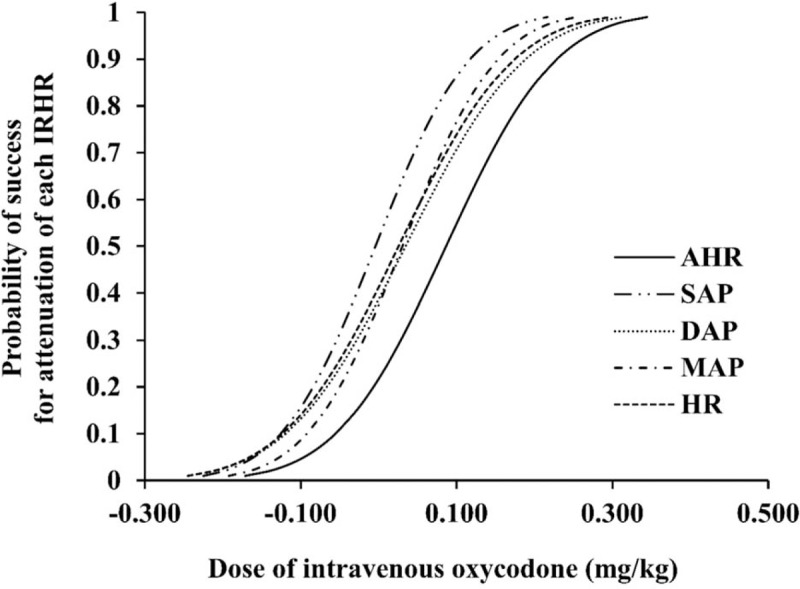
Dose-response curve plotted from probit regression analysis in “Part 2.” The recalculated oxycodone ED_95_ was 0.269, 0.152, 0.188, and 0.217 mg/kg for attenuating changes of AHR, SAP, DAP, MAP, and HR, respectively. AHR = all hemodynamic responses, DAP = diastolic arterial pressure, HR = heart rate, IRHR = intubation-related hemodynamic response, MAP = mean arterial pressure, SAP = systolic arterial pressure.

Probit analysis was performed to derive the optimal dose in the oxycodone and normal saline groups (Fig. 7). We found that oxycodone 0.269 mg/kg was needed to prevent any arterial pressure and HR changes of more than 20% over the baseline value in 95% of the patients (ED_95_), which was derived from the oxycodone data in this randomized controlled study. Recalculated oxycodone ED_95_ values were 0.152, 0.232, 0.188, and 0.217 mg/kg for attenuating changes of SAP, DAP, MAP, and HR, respectively.

## 4 Discussion

We demonstrated that the predictive intravenous oxycodone ED_95_, which was defined as a dose for attenuating all IRHR changes to less than 20% over baseline values in 95% of the patients, was 0.091 mg/kg in male patients. Oxycodone 2 × ED_95_ (0.182 mg/kg) was more effective in preventing significant IRHRs than fentanyl 2 μg/kg, with less changes in arterial pressures and HR.

There are a few studies that used oxycodone doses of 0.1 or 0.2 mg/kg, which are similar to the doses of oxycodone ED_95_ (0.091 mg/kg) and 2 × ED_95_ (0.182 mg/kg) in this study, to determine the effective or optimal dose of oxycodone to minimize IRHRs.^[7–9]^ IRHR changes in these previous studies showed striking similarities to this study, increasing immediately after intubation and subsequently declining progressively. Intravenous oxycodone above 0.1 mg/kg was more effective than oxycodone below 0.1 mg/kg to maintain arterial pressures at 1 minute after intubation with non-significant differences.^[7,8]^ Oxycodone 0.14 mg/kg and 0.2 mg/kg showed an attenuating effect on arterial pressures comparable with fentanyl 2 μg/kg, which is known to be preferable to minimize the IRHRs in patients without hypertension.^[8,9,14]^ However, the HR after intubation remained significantly higher than the baseline values at all measured points, even though patients received either oxycodone or fentanyl.^[7,8]^ Lee at al^[9]^ reported that the maximal change of HR from the baseline value was not significantly different between oxycodone (0.2 mg/kg) and fentanyl (2 μg/kg) groups 5 minutes after following intubation, but the maximal changes above 20% was higher in the group receiving oxycodone (53%) than the group receiving fentanyl (47%). Bostan and Eroglu^[15]^ suggested that esmolol 1 mg/kg might provide reliable protection against increases in both HR and SAP compared with fentanyl 1 μg/kg. In this case, the addition of a β adrenergic blocker would be helpful to prevent intubation-related tachycardia if oxycodone of less than 0.2 mg/kg was used.^[7]^ Korpinen et al^[16]^ showed that esmolol (a β adrenergic blocker) 2 mg/kg before induction was effective in mitigating HR increase 1 minute after intubation in patients with a premedication of intravenous oxycodone 0.1 mg/kg 1 hour before anesthesia.

There has been no report on intravenous oxycodone ED_95_ using a less than 20% change as a cut-off level of all IRHRs. Recently, Park et al^[7]^ reported oxycodone ED_95_ with less than 15% changes as a cut-off level for individual IRHRs. They reported that oxycodone ED_95_ for attenuating the changes of SAP and MAP were 0.159 (95% CI, 0.122–0.243) mg/kg and 0.219 (95% CI, 0.171–0.335) mg/kg, respectively. However, oxycodone ED_95_ for preventing HR change could not be calculated, and they suggested that it was far higher than their experimental dose when it was calculated using a less than 20% change cut-off level.

This study showed that the predictive oxycodone ED_95_ was 0.091 (95% CI, 0.081–0.149) mg/kg in male patients using a less than 20% change cut-off level for all IRHRs. However, the oxycodone ED_95_ and 2 × ED_95_ showed actual preventing effects at rates of 62.2% and 75.7%, respectively. Even though these actual attenuating effects did not reach 95%, those of oxycodone 0.182 mg/kg (2 × ED_95_) increased to 94.6% and 89.2% for attenuating changes of SAP and MAP to less than 20%, respectively. The recalculated oxycodone ED_95_, which was derived oxycodone and normal saline group data, was 0.152 and 0.188 mg/kg for attenuating changes of SAP and MAP, respectively, which were within 95% CI of the results of Park study.^[7]^ In addition, the recalculated oxycodone ED_95_ was 0.269 mg/kg for attenuating all IRHRs, and 0.217 mg/kg for attenuating changes of HR.

Oxycodone as high as 0.269 mg/kg might be used in order to completely eliminate a significant increase in all IRHRs considered in this study, but complications such as hypotension, bradycardia, and respiratory depression would have to be considered at this higher dose of oxycodone. The incremental dosage of oxycodone up to 0.2 mg/kg showed significantly less changes of IRHRs with more suppressed trends compared to those seen with lower doses of oxycodone or fentanyl.^[7,8]^ Even though the hemodynamic complications of oxycodone up to 0.2 mg/kg have been reported to be comparable to those of fentanyl (2 μg/kg), the incidence of both hypotension (systolic blood pressure below 90 mm Hg) and bradycardia (HR below 60 beats/min) was about 33%.^[8,9]^ Our results for IRHRs were also similar to those of previous reports.^[7–9]^ In this study, we did not observe any significant hypotension and bradycardia after administration of oxycodone (0.091 mg/kg and 0.182 mg/kg) because it was administered slowly over 2 minutes. The administration of oxycodone can induce a significantly higher incidence of desaturation compared with that of fentanyl in patients without pre-oxygenation.^[9]^ However, we did not observe significant desaturation because all patients were pre-oxygenated.

Various doses of fentanyl (1 μg/kg, 1.5 μg/kg, 2 μg/kg, 3 μg/kg, 5 μg/kg) were used to study the effect on preventing morbidity associated with IRHRs.^[4]^ Kautto^[17]^ reported that 2 μg/kg of fentanyl 3.5 minutes before endotracheal intubation only decreased HR but larger doses of fentanyl decreased both HR and blood pressure. In our hospital, we commonly use 2 μg/kg of fentanyl for preventing IRHRs. Oxycodone has been reported to be effective for controlling preoperative acute pain such as bone fracture,^[18]^ and preoperative administration was associated with the beneficial effect of reducing postoperative opioid consumption without an increase in side effects.^[19]^ Therefore, we assumed that intravenous oxycodone also could be used effectively to attenuate IRHRs, and we chose fentanyl 2 μg/kg and intravenous oxycodone for this study.

The onset and action duration of fentanyl is 2 to 3 minutes and 3 hours 39 minutes, respectively.^[7,20]^ While intravenous oxycodone has an onset similar to that of fentanyl, it has a longer action duration (4 hours 52 minutes) than that of fentanyl.^[7,20]^ The peak effect of fentanyl and oxycodone is achieved within 5 minutes and 15 to 30 minutes after intravenous injection, respectively.^[21,22]^ Thus, we administrated fentanyl and oxycodone at 5 and 20 minutes before anesthesia induction, respectively, with consideration of the patient's safety and peak effect time.

There are some limitations associated with the present study. First, the enrolled patients were all male adults aged 20 to 65 years. The individual titration of oxycodone dosage is important, particularly in older adults. Liukas et al^[10]^ reported that age is a key factor affecting the pharmacokinetics of oxycodone in older patients aged >70 years. Sex also is a contributor to differences in oxycodone serum concentration, which is lower in women than in men.^[11]^ Second, we did not exclude patients using routine opioids preoperatively, which can alter IRHRs. However, none of these patients were enrolled in this study. Third, all patients were premedicated with intramuscular midazolam. Intramuscular midazolam can influence IRHRs with stabilized hemodynamics and an analgesic effect during the induction of anesthesia.^[23]^ However, we ignored this effect in our study because it is a positive effect on IRHRs and we use intramuscular midazolam routinely as an anxiolytic, except where contraindicated. Fourth, we did not record the Mallampati grades of all patients. If patients had high Mallampati grades, intubation within 15 seconds would have failed, and we excluded these patients in the final statistical analysis. Fifth, a relatively small sample size was used for calculating the predictive oxycodone ED_95_ in part 1. The sample size was enough for the up-and-down methods, but the small sample size might have led to wide range of confidence intervals. Therefore, the predictive oxycodone ED_95_ showed a 75.7% “success” rate in part 2, and the recalculated oxycodone ED_95_ with probit analysis (0.269 mg/kg) was needed to prevent any arterial pressure and HR changes. Finally, we did not evaluate intraoperative hemodynamics and postoperative effects regarding emergent or postoperative pain. Oxycodone may affect intraoperative hemodynamics as well as the intraoperative and postoperative requirement for opioids, because the onset time of oxycodone and duration of action of intravenous oxycodone are about 3 minutes and 4 hours, respectively.^[9,19,24]^ Therefore, further research is required to obtain the predictive ED_50_ and ED_95_ of oxycodone-based on age and sex, and to investigate the intraoperative and postoperative effects on hemodynamics and the analgesic requirements using these doses of oxycodone. In addition, further research is required to determine whether the recalculated oxycodone ED_95_ (0.269 mg/kg) for preventing all IRHRs is also effective and has hemodynamic safety with larger sample size.

In conclusion, the predictive intravenous oxycodone ED_95_, for attenuating all changes of hemodynamic responses to less than 20%, was 0.091 mg/kg in male patients. The administration of oxycodone 0.182 mg/kg (2 × ED_95_) 20 minutes before intubation was more effective in attenuating IRHRs than fentanyl 2 μg/kg 5 minutes before intubation, and resulted in smaller hemodynamic changes. However, oxycodone 0.182 mg/kg (2 × ED_95_) was not sufficient to blunt increases in HR after intubation; oxycodone above 0.2 mg/kg, or a combination with a β adrenergic blocker would be required. In addition, we recommend that slow injection and pre-oxygenation should be performed after the administration of oxycodone to minimize the risk of complications before intubation. Moreover, our results provide guidelines for preoperative intravenous oxycodone use and its reference dosage in patients requiring control acute pain before surgery. Oxycodone may provide effective preoperative pain control as well as attenuation of IRHRs without the addition of other analgesics for these purposes in patients who received intravenous oxycodone preoperatively.

## Author contributions

**Conceptualization:** Ki Tae Jung, Keum Young So, Sang Hun Kim.

**Data curation:** Ki Tae Jung, Jong Sik Seo, Sang Hun Kim.

**Formal analysis:** Gi-Ho Koh, Ki Tae Jung, Keum Young So, Jong Sik Seo, Sang Hun Kim.

**Investigation:** Ki Tae Jung, Keum Young So, Jong Sik Seo, Sang Hun Kim.

**Methodology:** Sang Hun Kim.

**Project administration:** Ki Tae Jung, Keum Young So, Sang Hun Kim.

**Resources:** Sang Hun Kim.

**Software:** Gi-Ho Koh, Sang Hun Kim.

**Supervision:** Ki Tae Jung, Keum Young So, Sang Hun Kim.

**Validation:** Gi-Ho Koh, Sang Hun Kim.

**Visualization:** Ki Tae Jung, Sang Hun Kim.

**Writing – original draft:** Gi-Ho Koh.

**Writing – review and editing:** Ki Tae Jung, Keum Young So, Jong Sik Seo, Sang Hun Kim.

Sang Hun Kim orcid: 0000-0003-3869-9470.
